# A familial case of Kikuchi-Fujimoto disease in dizygotic twins

**DOI:** 10.1186/s12969-020-00457-2

**Published:** 2020-08-10

**Authors:** Ashfaque Quadir, Ken Peacock, Peter Hsu, Davinder Singh-Grewal, Stephen Alexander

**Affiliations:** 1 Department of General Medicine, The Children’s Hospital at Westmead, Westmead, Sydney, NSW 2145 Australia; 2 Department of Immunology, The Children’s Hospital at Westmead, Westmead, Sydney, NSW 2145 Australia; 3 Department of Rheumatology, The Sydney Children’s Hospitals Network, Westmead and Randwick, Sydney, NSW 2145 Australia; 4 Department of Nephrology, The Children’s Hospital at Westmead, Westmead, Sydney, NSW 2145 Australia

**Keywords:** Kikuchi-Fujimoto, Twins, Familial, Lymphadenopathy, Necrotizing, Histiocytic, Lymphadenitis

## Abstract

**Background:**

Kikuchi-Fujimoto disease (KFD) or necrotizing histiocytic lymphadenitis, was described separately by both Kikuchi and Fujimoto in Japan in the early 1970’s. Despite its rarity in the pediatric population, it is an important differential in persistent lymphadenopathy. Familial cases of KFD in the literature are rare. Here we describe the first reported case of KFD in non-identical twin sisters.

**Case presentation:**

Twin 1 presented with a 3-week history of worsening right-sided cervical lymphadenopathy, daily fevers, significant lethargy, weight loss and arthralgia of her knees and ankles at the age of 12 years in 2015. She had had an unremarkable medical history. A biopsy of her lymph nodes showed histiocytic necrosis consistent with KFD. Twin 2 presented with a three-week history of lethargy, fatigue, weight loss and left-sided posterior cervical chain lymphadenopathy at 16 years of age in 2018. She had a history of frequently relapsing nephrotic syndrome and celiac disease. A biopsy of her lymph nodes was undertaken and showed histiocytic necrosis consistent with KFD.

**Conclusions:**

KFD is a rare but self-limiting pathological process of necrotizing histiocytic lymphadenitis. Although further research is needed, there is an increasing amount of evidence which suggests a multifactorial pathological basis of disease. The two cases we document here are the first reported cases of familial KFD in dizygotic HLA-identical twins which reinforces the likely HLA-linkage in the etiology of KFD.

## Background

Kikuchi-Fujimoto disease (KFD) or necrotizing histiocytic lymphadenitis, was described separately by both Kikuchi and Fujimoto in Japan in the early 1970’s [1, 2]. Despite its rarity in the pediatric population, it is an important differential in persistent lymphadenopathy. KFD occurs predominantly in young women with associated symptoms of fever, lethargy, arthralgia and rash. Although the current etiology of KFD is unclear, associations have been made with autoimmune disease; with various infectious triggers including several viruses and other non-infective inflammatory conditions suggested. Familial cases of KFD in the literature are rare. Here we describe the first reported case of KFD in non-identical twin sisters.

## Case presentation

### Twin 1

Twin 1 presented at the age of 12 years in 2015. She had had an unremarkable medical history although there was significant family history which is outlined in our second case. The family were of Lebanese descent. She presented with a 3-week history of worsening right-sided cervical lymphadenopathy, daily fevers, significant lethargy, weight loss and arthralgia of her knees and ankles. On examination, she had firm enlarged lymph nodes in the submandibular, posterior chain and supraclavicular regions predominantly on the right but also on the left side of her neck. There was no overlying erythema or cutaneous change noted. There was also no significant joint swelling or effusion.

She was initially treated with intravenous antibiotics with little improvement. A biopsy of her right sided lymph nodes was undertaken and showed histiocytic necrosis consistent with KFD. Her fevers resolved during her hospital admission although her lymphadenopathy persisted. A day prior to her discharge from hospital she developed an erythematous maculopapular rash over her face, back and limbs.

Over the following months, she developed alopecia areata, arthralgia and oral ulceration with persistent and severe fatigue. Her rash subsided without intervention. She was trialed on a short course of oral steroids and her symptoms slowly resolved. Twin 1 after 2 years of follow-up showed no serological evidence of Systemic Lupus Erythematosus or other autoimmune disease and was well and attending school.

### Twin 2

The second twin presented at 16 years of age in 2018. She had a history of frequently relapsing nephrotic syndrome from the age of 4. She required treatment with corticosteroids until the age of 12 along with other immunosuppressive medications including Cyclosporin, Tacrolimus and Mycophenolate Mofetil. She was subsequently commenced on intermittent Rituximab infusions leading to remission and prior to presentation with her lymphadenopathy had been in remission for more than 2 years. Complications of her treatment included steroid-induced osteoporosis which required intermittent intravenous bisphosphonate therapy. She had also been diagnosed with celiac disease treated with a gluten-free diet and was noted to be a CYP2C19 intermediate metaboliser.

She presented with a three-week history of lethargy, fatigue, weight loss and left-sided posterior cervical chain lymphadenopathy. Fever commenced 2 days prior to her presentation with associated night sweats and a 2-day history of coryzal symptoms. On examination, she had extensive lymphadenopathy of her left posterior cervical chain with no rash, joint changes or hepatosplenomegaly.

She was initially commenced on intravenous antibiotics. Her serology was positive for Mycoplasma and her nasopharyngeal aspirate was PCR positive for Respiratory Syncitiovirus. A biopsy of her lymph nodes was undertaken and showed histiocytic necrosis consistent with KFD.

After a one-week admission, she was discharged when her fevers had resolved. She subsequently developed arthralgia and a rash similarly to her sister but had no serological evidence of SLE. She was commenced on a course of oral steroids with subsequent improvement in her symptoms over time.

### Investigations

**Fig. 1:**
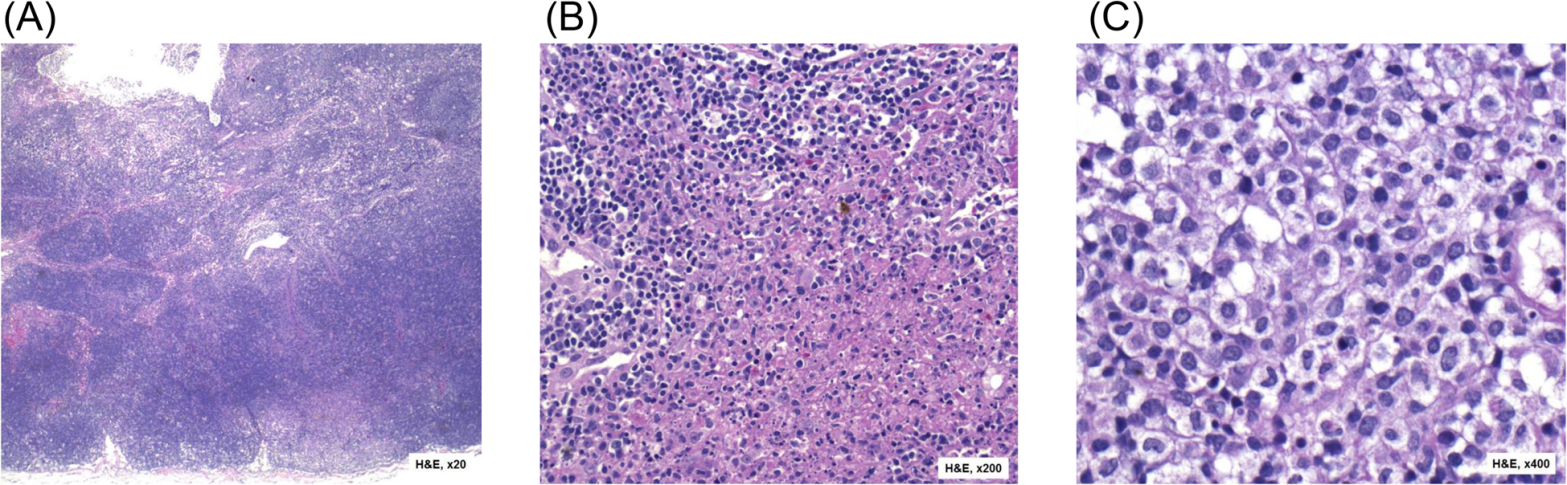
Histopathology slides taken from a lymph node biopsy of Twin 2. Stained with Haematoxylin and Eosin with magnification as noted in the images. **a** – Loss of the normal follicular architecture of the lymph node with an area of abnormal cellularity in the subcapsular area. **b** – Abrupt coagulative necrosis with no neutrophils (“sterile” necrosis). **c** – Epithelioid cells with abnormal morphology which are the cells of origin for the coagulative necrosis

**Fig. 2:**
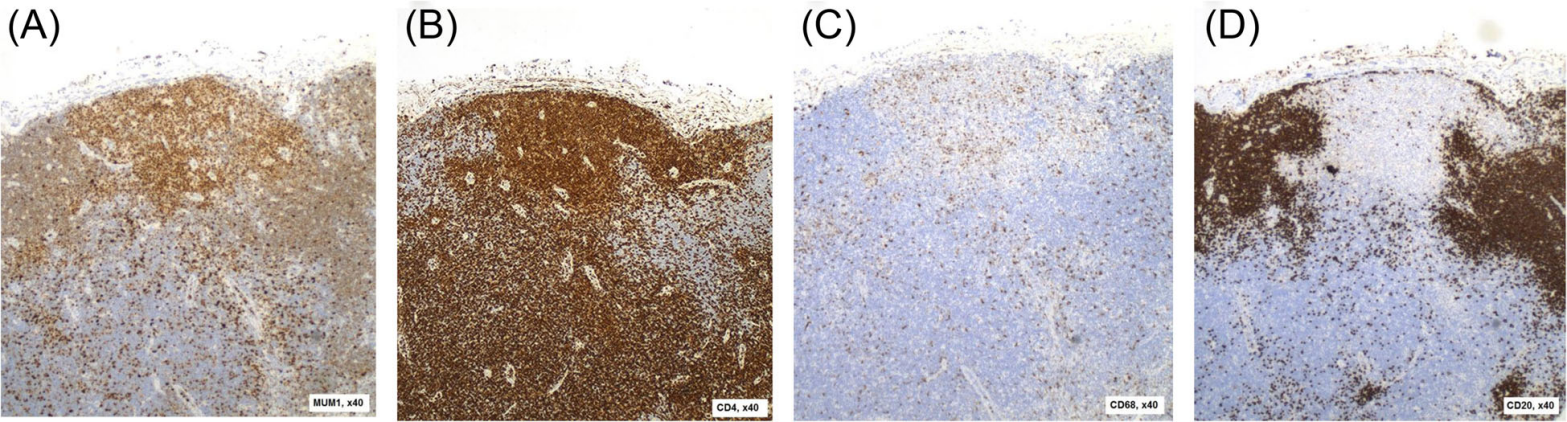
Histopathology slides taken from a lymph node biopsy of Twin 2 with immunohistochemical staining for various cell surface markers. **a** – Positive staining for MUM1 (general lymphocyte activation marker). **b** – Positive staining for CD4 (T-cell marker). **c** – Positive staining for CD68 (histiocyte marker). **d** – Negative staining for CD20 (B-cell marker). This shows the abnormal population of epithelioid cells which are positive for T-cell and hystiocyte markers and negative for B-cell markers and thus are the blastic T-cells that proliferate in Kikuchi-Fujimoto disease

Both twins were comprehensively investigated with serological and biochemical testing as well as imaging, lymph node biopsy and tissue typing (Table 1). The twins were HLA identical on examination of 8 HLA loci. Histology from Twin 2’s biopsy is shown in Figs. 1 and 2. They demonstrate loss of the lymph node’s regular follicular architecture and areas of nonsuppurative necrosis. There is also a population of abnormal epithelioid cells which are positive for T-cell and histiocyte markers and negative for B cell markers. These are proliferating blastic T-cells that are found in Kikuchi-Fujimoto disease.

**Table 1:** Investigation Results for Twin 1 and Twin 2

Test		Twin 1	Twin 2	Normal Range
Full Blood Count	Haemoglobin	106 (L)	104 (L)	115–150 g/L
	White cell count	3.4 (L)	3.6 (L)	4.5–13.5 × 10^9/L
	Platelet count	199	262	150–600 × 10^9/L
	Neutrophil count	1.5	1.9	1.5–8.0 × 10^9/L
	Lymphocyte count	1.5	1.4	1.0–4.0 × 10^9/L
Liver Function Test		Within normal limits	Within normal limits	
Electrolytes/Urea/Creatinine		Within normal limits	Within normal limits	
C-reactive Protein		60 (H)	82.2 (H)	0–10 mg/L
Erythrocyte Sedimentation Rate		101 (H)	50 (H)	0–20 mm/hr
ANA screen		Negative	Negative	
ENA screen		Not detected	Not detected	
Anti-dsDNA antibodies		Negative	Negative	
Complement C3		1.68	1.43	0.75–1.75 g/L
Complement C4		0.34	0.32	0.13–0.52 g/L
Urate		0.24	0.30	0.14–0.36 mmol/L
Lactate Dehydrogenase		586	266 (L)	313–618 U/L
Immunoglobulins	IgG	N/A	4.33 (L)	6.24–14.40 g/L
	IgA	N/A	< 0.06 (L)	0.59–3.96
	IgM	N/A	0.73	0.48–3.04
	IgE	N/A	< 20	0–200 IU/mL
Toxoplasma serology		IgM/IgG negative	IgM/IgG negative	
EBV serology		IgM/IgG negative	IgM/IgG negative	
Other Infective serology		Nil	Mycoplasma IgM positive, RSV PCR positive	
Histopathology		Histiocytic necrosis, no bacteria on gram stain, no AFB on ZN, no fungal elements on PAS	Histiocytic necrosis, no bacteria on gram stain, no AFB on ZN, no fungal elements on PAS, flow cytometry showed no abnormal cell population	
Imaging		CT: Extensive cervical lymphadenopathy with no other focus	Ultrasound: Multiple enlarged lymph nodes in the left cervical chain with increased vascularity, largest measures 20 × 13 mm	
HLA Class I	A	02:01, 03:02	02:01, 03:02	
	B	08:01, 35:08	08:01, 35:08	
	C	04:01, 07:02	04:01, 07:02	
HLA Class II	DRB1	03:01, 07:01	03:01, 07:01	
	DRB3	02:02	02:02	
	DRB4	01:03	01:03	
	DPB1	04:01, 23:01	04:01, 23:01	
	DQB1	02:01, 02:02	02:01, 02:02

## Discussion

Kikuchi-Fujimoto disease remains more of a syndrome than a fully characterized disease. Twin 2 had nephrotic syndrome, a disease in which there is HLA linkage and evidence of viral activation similar to KFD [3]. The presence of mycoplasma with positive mycoplasma serology at the time of diagnosis in Twin 2 would be consistent with an infectious trigger for KFD [4].

Although very limited in number, there have been previous case reports documenting familial cases of KFD in siblings and even HLA-identical non-twin sisters [5–7]. Some of these familial cases have been documented in Japanese literature where related individuals who developed KFD were living in the same environment, were serologically positive for the same infectious trigger and had developed symptoms within a short time period of each other. Exceptions to this include the case report by Amir et al. (2002) in Saudi Arabia where two sisters who were HLA-identical but not twins had presented 10 years apart with no obvious infectious trigger identified and the article by Stasiuk et al. (2011) which reported a similar case in two Aboriginal sisters from Northern Ontario [5, 6]. The shared and common HLA for this disease suggests that like many other HLA-linked diseases, KFD is a two-step disease requiring a predisposing HLA and a secondary trigger such as an infection. Twin 2’s background is particularly interesting as she has two other diseases that are linked to HLA Class II with celiac disease and nephrotic syndrome. This would be consistent with the paradigm that KFD needs an infectious trigger to develop but requires a specific HLA to develop disease. Although multiple offenders have been identified including Epstein Barr Virus, various Human Herpes Viruses, HIV, Torque Teno virus and Toxoplasma Gondii, it remains largely unclear if these pathogens are etiological or more likely inflammatory drivers of disease through TLR or other innate receptors [4, 8–10].

Of note, the HLA typing of the sets of siblings described in Stasiuk et al. and Amir et al.’s case reports are shown in Table 2 [5, 6]. Interestingly, our twins’ HLA-C type was identical to the siblings reported by Amir et al.^6^. Although the significance of this is unclear, this further supports the hypothesis regarding the etiology of KFD that we have put forward in this report.

**Table 2:** Comparison of HLA-Typing in Siblings with Kikuchi-Fujimoto Disease

		Twin 1	Twin 2	Sibling 1 (Stasiuk et al)^5^	Sibling 2 (Stasiuk et al)^5^	Sibling 1 (Amir et al)^6^	Sibling 2 (Amir et al)^6^
HLA Class I	A	02:01, 03:02	02:01, 03:02	02	02, 31	31	31
	B	08:01, 35:08	08:01, 35:08	39, 35	39, 51	35, 49	35, 49
	C	04:01, 07:02	04:01, 07:02			04, 07	04, 07
	BW					04, 06	04, 06
	Cw			07, 04	07, 15		
HLA Class II	DRB1	03:01, 07:01	03:01, 07:01	08, 14	08, 14		
	DRB3	02:02	02:02				
	DRB4	01:03	01:03				
	DPB1	04:01, 23:01	04:01, 23:01				
	DR					15, 13	15, 13
	DRW					51, 52	51, 52
	DQ					06	06
	DQA1	02:01, 05.01	02:01, 05.01				
	DQB1	02:01, 02.02	02:01, 02.02	03	03, 0402		

There have been many associations with KFD and other diseases in various case reports in the literature including but not exclusive to hemolytic uremic syndrome, Hashimoto’s thyroiditis, hemophagocytic lymphohistiocytosis and more commonly, systemic lupus erythematosus [11–15]. Twin 2 had a background of nephrotic syndrome and celiac disease as discussed above. Both twins developed mucocutaneous symptoms that led to investigation for SLE but yielded no positive results. Although unclear, these associations with autoimmune conditions should guide clinicians when monitoring patients with prior KFD particularly in the context of disease surveillance or in the presence of new symptoms.

Interestingly, Twin 2 was serologically positive for *Mycoplasma pneumoniae* of which an association with KFD has not previously been documented. Although this could be coincidental, it should be noted that both Mycoplasma and KFD can lead to clinical signs and symptoms that may mimic other conditions or cause atypical presentations.

## Conclusion

KFD is a rare but self-limiting pathological process of necrotizing histiocytic lymphadenitis. Although further research is needed, there is an increasing amount of evidence which suggests a multifactorial pathological basis of disease. The two cases we document here are the first reported cases of familial KFD in dizygotic HLA-identical twins which reinforces the likely HLA-linkage in the etiology of KFD. Due to its ability to mimic other more sinister illnesses, recognition and appropriate diagnosis of KFD is paramount to ensure that unnecessary investigations and treatment are avoided.

## Data Availability

Not applicable.

## Abbreviations

KFD: Kikuchi-Fujimoto Disease
PCR: Polymerase Chain Reaction
SLE: Systemic Lupus Erythematosus
HLA: Human Leukocyte Antigen
HIV: Human Immunodeficiency Virus
TLR: Toll-like Receptors
